# Development of an oxidative stress *in vitro* assay in zebrafish (*Danio rerio*) cell lines

**DOI:** 10.1038/s41598-018-30880-1

**Published:** 2018-08-17

**Authors:** Sebastian Lungu-Mitea, Agneta Oskarsson, Johan Lundqvist

**Affiliations:** Department of Biomedicine and Veterinary Public Health, Swedish University of Agricultural Sciences, Box 7028, SE-750 07 Uppsala, Sweden

## Abstract

The nuclear factor erythroid 2-related factor 2 (Nrf2) is a key regulator of cellular defense against oxidative stress and correlated with classical toxicological endpoints. *In vitro* methods using fish cell lines for the assessment of aquatic toxicity are needed for mechanistic studies and as an alternative to *in vivo*. We describe an *in vitro* assay to study oxidative stress using zebrafish cell lines. Transfection efficiency of twelve commercially available transfection reagents were tested in the zebrafish cell lines ZFL, ZF4, and Pac2. The most efficient reagent for each cell line was selected for further experiments. Cells were transiently transfected with an Nrf2-responsive luciferase plasmid. The assay was tested using the oxidative stress inducing chemicals *tert*butylhydroquinone, hydrogen peroxide, and sulforaphane. Of the transfected cell lines, ZF4 and ZFL showed higher sensitivity. The latter were used to study potential oxidative stress induced by pesticides (diazinon, deltamethrin, atrazine, metazachlor, terbutylazine, diuron). Besides known inducers, Nrf2 activity was also significantly induced by diazinon, deltametrin, diuron, and metazachlor. Activation of Nrf2 by metazachlor is a novel finding. The described assay could be a valuable tool for research in toxicology to study the stress response of both pure chemicals and environmental water samples.

## Introduction

Oxidative stress, with formation of reactive oxygen species (ROS), is a mechanism underlying a number of toxicity processes^1^. The nuclear factor erythroid 2-related factor 2 (Nrf2) is a keystone in the cellular defense against oxidants and the regulation of oxidative stress detoxification, metabolism, and induction of related phase-II enzymes (glutathione S-transferase (GST), NADPH-quinone oxireductase 1 (NQO1), heme oxygenase 1 (HO-1), g-glutamylcysteine synthetase (g-GCS), thioredoxin (TRX))^2^. The antioxidant responsive element (ARE) sequence (“RTGACnnnGC”)^3,4^ is located upstream in the regulatory region of the phase-II metabolizing enzymes genes, often coinciding with the xenobiotic response element (XRE)^5^. Nrf2 occurs ubiquitously under normal physiological conditions in the cytosol where it is bound to its negative regulator Kelch-like ECH-associated protein 1 (Keap1), which marks it for degradation^6^. Upon conditions of oxidative stress Nrf2 is released from Keap1 and avoids proteolysis. Nrf2 then translocates and accumulates within the nucleus where it heterodimerizes with small Maf proteins and initiates gene expression^7^ by binding to AREs. The Keap1-Nrf2-ARE signaling pathway is highly conserved within vertebrates^8–10^. In ToxCast (“toxicity forecaster”, an online database by the US Environmental Protection Agency, USEPA, https://www.epa.gov/chemical-research/toxcast-dashboard) high-throughput data on toxicity, *e.g.* Nrf2 activity, of single chemicals are collected. Nrf2 activity has been shown to be correlated with classical toxicological endpoints *in vivo* and it has therefore been suggested that oxidative stress response and Nrf2 activity would be an ideal pathway to build *in vitro* toxicity assays on^11^.

*In vivo* assays are extensively used in assessment of aquatic toxicity of chemicals. The high number of animals used in environmental risk assessment has highlighted the need for novel approaches and development of *in vitro* assays. Development of fish cell culture-based *in vitro* assays has been proposed as a promising idea to reduce and replace the use of fish in aquatic toxicity testing, *e.g*. by the European Union Reference Laboratory for Alternatives to Animal Testing (EURL ECVAM)^12–14^. Development of such *in vitro* models would allow subsequent high throughput screening and application of omics technologies. Cell cultures and embryo tests have emerged as useful alternative approaches in environmental toxicology^15^.

Reporter gene assays in transfected mammalian cells have been shown to be a valuable tool for research in many fields of toxicology^16–19^. However, the use of fish cell lines for reporter gene assays has been very limited and this type of approach is still in its infancy within aquatic toxicology. A requirement for the development of reporter gene assays, is the availability of functioning transfection methods. Most transfection reagents were developed for mammalian cell lines and are based on lipid-fusion mechanisms. However, the latter might be less efficient in fish cells due to lower incubation temperatures^20^. Furthermore, the transfection efficiency for a specific transfection regent often varies greatly between cell lines^21^. Thus, in the present investigation cells of different developmental stages and tissue origin were tested.

In this study, we have established a reporter gene assay for detecting oxidative stress by measuring induction of free Nrf2 in zebrafish cell lines Pac2, ZF4, and ZFL. Initially, we tested the transfection efficiency of twelve commercially available transfection reagents for these three cell lines. The established reporter gene assays were tested with potential Nrf2 inducers and six pesticides which are suspected to cause oxidative stress^22^. The established *in vitro* bioassay might be a useful tool in screening for potential inducers of oxidative stress, both regarding toxicity of pure compounds and for analysis of environmental samples, *e.g*. for ecotoxicology and environmental monitoring.

## Materials and Methods

### Cell culture

#### Zebrafish fibroblast cell line Pac 2

Zebrafish fibroblast cell line Pac 2^23,24^ (CVCL_5853) was cultured in Leibovitz’s L-15 medium containing phenol red (Sigma-Aldrich, Steinheim, Germany), supplemented with 15% fetal bovine serum (Gibco, Paisley, UK), 1% penicillin-streptomycin 100 U/mL (Gibco, Paisley, UK), 1‰ gentamicin 50 mg/mL (Lonza, Basel, Swiss). The cells were cultured in a humidified environment at 28 °C and atmospheric CO_2_. The cells were passaged weekly in a 1 to 3 ratio, using PBS (Medicago, Uppsala, Sweden) for washing and 0.25% Trypsin-EDTA (Sigma-Aldrich, Steinheim, Germany) for detachment.

#### Zebrafish fibroblast cell line ZF4

Embryonic zebrafish fibroblast cell line ZF4^25^ (CVCL_3275) was cultured in Dulbecco’s modified Eagle’s medium/Ham’s Nutrient Mixture F-12 (DMEM:F12) containing phenol red (Gibco, Paisley, UK), supplemented with 10% fetal bovine serum (Gibco, Paisley, UK), 1% penicillin-streptomycin 100 U/mL (Gibco, Paisley, UK), 2.5 mM L-glutamine (Lonza, Basel, Swiss), 15 mM HEPES (Gibco, Paisley, UK), 0.5 mM sodium pyruvate (Sigma-Aldrich, Steinheim, Germany), and 1200 mg/L sodium bicarbonate (Gibco, Paisley, UK). The cells were cultured in a humidified environment at 28 °C and with 5% CO_2_. The cells were passaged every 3 to 4 days in a 1 to 3 ratio, using PBS (Medicago, Uppsala, Sweden) for washing and 0.25% trypsin (Sigma-Aldrich, Steinheim, Germany) for detachment.

#### Zebrafish liver cell line ZFL

Zebrafish liver cell line ZFL^26,27^ (CVCL_3276) was cultured in a medium consisting of 50% Leibovitz’s L-15 (Sigma-Aldrich, Steinheim, Germany), 35% Dulbecco’s modified Eagle’s medium (Gibco, Paisley, UK), 15% Ham’s Nutrient Mixture F-12 (Gibco, Paisley, UK), and phenol red. Additionally, 150 mg/L sodium bicarbonate (Gibco, Paisley, UK), 15 mM HEPES (Gibco, Paisley, UK), 10 µg/mL bovine insulin (Sigma-Aldrich, Steinheim, Germany), 50 ng/mL mouse EGF (Sigma-Aldrich, Steinheim, Germany), and 5% fetal bovine serum (Gibco, Paisley, UK) were supplemented. The cells were cultured in a humidified environment at 28 °C and atmospheric CO_2_. The cells were passaged every 3 to 4 days in a 1 to 4 ratio, using PBS (Medicago, Uppsala, Sweden) for washing and 0.25% Trypsin-EDTA (Sigma-Aldrich, Steinheim, Germany) for detachment.

### Plasmid

The pGL4.37[luc2P/ARE/Hygro] plasmid was acquired from Promega, Madison, USA. The pGL4.37[luc2P/ARE/Hygro] vector consists of a pGL4 backbone including an ampicillin resistance gene, a gene for hygromycin resistance, and four copies of an Nrf2-sensitive antioxidant response element (ARE) driving transcription of the luciferase reporter gene luc2P (*Photinus pyralis*). A plasmid including the cDNA (Rluc) encoding Renilla luciferase reporter gene (*Renilla reniformis*) was used as a control of transfection efficiency and for normalization.

### Chemicals

The following chemicals were used for exposure studies: atrazine (CAS 1912-24-9), 99.3% purity, Dr. Ehrenstorfer GmbH, Augsburg, Germany; deltamethrin (CAS 52918-63-5), 99.5% purity, Dr. Ehrenstorfer GmbH, Augsburg, Germany; diazinon (333-41-5), 99% purity, Dr. Ehrenstorfer GmbH, Augsburg, Germany; Dimethyl sulfoxide (CAS 67-68-5), 99.9% purity, Sigma-Aldrich, St. Louis, USA; diuron (CAS 330-54-1), 98.8% purity, Dr. Ehrenstorfer GmbH, Augsburg, Germany; hydrogen peroxide (H_2_O_2_) (CAS 7722-84-1), Sigma-Aldrich, Steinheim, Germany; metazachlor (CAS 67129-08-2), 99.5% purity, Dr. Ehrenstorfer GmbH, Augsburg, Germany; sulforaphane (SFN) (CAS 4478-93-7), 90% purity, Sigma-Aldrich, Steinheim, Germany; terbutylazine (tBA) (CAS 5915-41-3), 99.5% purity, Dr. Ehrenstorfer GmbH, Augsburg, Germany; *tert*butylhydroquinone (tBHQ) (CAS 1948-33-0), 97% purity, Sigma-Aldrich, St. Louis, USA.

The compounds were dissolved in 20 mM stock solutions of atrazine, deltamethrin, diazinon, diuron, metazachlor, and terbutylazine in 99% EtOH and stored at −20 °C. Stock solutions of H_2_O_2_ and tBHQ of 100 mM were prepared in 99% EtOH and stored at −20 °C for every new experiment. A 50 mM stock of SFN in 99% EtOH was prepared and stored at −80 °C.

### Dual reporter gene assay

#### Transfection of zebrafish cell lines

Twelve commercially available transfection reagents: TransIT-X2®, TransIT®-LT1, TransIT®-2020 (Mirus, Madison, USA); FuGENE® HD (FHD), FuGENE® 6, ViaFect™, TransFast™ (Promega, Madison, USA); X-tremeGENE HP (XHP) (Roche, Mannheim, Germany); Lipofectamine® 2000 (Invitrogen, Carlsbad, USA); SuperFect®, Effectene® (Qiagen, Hilden, Germany); jetPRIME® (Polyplus, Illkirch-Graffenstaden, France) were tested for suitability to zebrafish cells and maximal induced transfection efficiency. Cells of all three mentioned cell lines were seeded at a density of 10^4^ cells/well in white, clear-bottom 96-well plates (Corning, New York, USA) in 100 µl/well. After 24 h incubation at above stated conditions, cells were transiently transfected with the Renilla plasmid only. Varying reagent to DNA mass ratios were applied in quadruplicates according to the manufacturer’s instructions. Mirus, Promega, Roche, and Invitrogen derived reagents were applied in a 1 µg DNA to 2, 4, or 6 µg reagent mass ratio, SuperFect in a 1 µg DNA to 1, 5, or 10 µg reagent mass ratio, Effectene in a 1 µg DNA to 10, 25, or 50 µg reagent mass ratio and jetPrime in a 1 µg DNA to 1, 2, or 3 µg reagent mass ratio. Total background Renilla luciferase induction for the single ratios in comparison to solvent control was considered as the benchmark. Subsequently, for most promising candidates, series of different cell concentrations (10^3^ cells/well, 5*10^3^ cells/well, 10^4^ cells/well, 5*10^4^ cells/well, 10^5^ cells/well, 5*10^5^ cells/well in 100 µl/well) were prepared and transiently transfected at the optimal ratio, in order to determine the best DNA-reagent-ratio to cell-density parameters. Induced background luminescence was scored identically to procedures described in the following section, with the only difference that a single luminescence signal was required.

#### Nrf2-responsive assay

Above mentioned cell lines were transiently co-transfected with the pGL4.37[luc2P/ARE/Hygro] plasmid and the Renilla plasmid using FHD (Promega, Madison, USA) for Pac2 and ZF4, and XHP (Roche, Mannheim, Germany) for ZFL. Subsequently, after exposure to known Nrf2 inducers or pesticides, Nrf2 induction was analyzed using a Dual-Luciferase® Reporter (DLR™) assay (Promega, Madison, USA). Cells were seeded into white, clear-bottom 96-well plates (Corning, New York, USA) at a density of 10^4^ cellls/well for Pac2, 1.25*10^4^ cells/well for ZF4, and 2.5*10^4^ cells/well for ZFL in 100 µl/well. After 24 h of incubation, cells reached a confluency of about 80%. Transient transfection using FHD or XHP, respectively was conducted in a 2 µg reagent to 1.2 µg DNA (0.9 µg reporter plasmid, 0.3 µg control plasmid) ratio, according to the manufacturer’s instructions. After 24 h of post-transfection incubation, cells were exposed to postulated Nrf2 inducers or pesticides. Prepared stock solutions (see section “Chemicals”) were further diluted using the cell type specific nutrition medium (see section “Cell culture”) supplemented with 5‰ EtOH as a solvent. Seeded cells on 96-well plates were either exposed in technical quadruplicates to increasing concentrations (0.1 µM, 1 µM, 10 µM, 100 µM) of postulated Nrf2 inducers tBHQ, SFN, and H_2_O_2_ or to increasing concentrations (6.25 µM, 12.5 µM, 25 µM, 50 µM, 100 µM) of the pesticides atrazine, deltamethrin, diazinon, diuron, metazachlor, and terbutylazine. Double quadruplicates of 5‰ EtOH solvent-nutrition medium were used as controls. In parallel with the oxidative stress exposure, increasing DMSO concentrations (1%, 4%, 7%, and 10% v/v) were tested as a cytotoxic control. After 24 h incubation, cells were lysed and quantitative Nrf2-dependent luminescence was measured via Dual-Luciferase® Reporter assay according to the manufacturer’s protocol using an auto-injecting Infinite M1000 microplate reader (Tecan, Männedorf, Switzerland). The luciferase activity was expressed as fold change compared to the controls.

### Cell viability testing

In order to determine cytotoxic concentrations of used compounds within the exposure range, cell viability was examined using an MTS-based [3-(4,5-dimethylthiazol-2-yl)-5-(3-carboxymethoxyphenyl)-2-(4-sulfophenyl)-2 H-tetrazolium] CellTiter 96® AQueous One Solution Cell Proliferation Assay (Promega, Madison, USA) in accordance with the manufacturer’s protocol. Viability tests were conducted in parallel to the dual reporter gene assays, applying the same arrangements and exposure set-ups. Cells were seeded in transparent 96-well plates (Corning, New York, USA) and transient transfection was omitted. Simultaneously to transfected cells being lysed for scoring of luminescence signal, nutrition medium of cells being used in viability test was discharged and replaced by PBS containing 17% (v/v) MTS-reagent. After 2 h of incubation, formazan product turnover absorbance was measured at 490 nm using a Wallac Victor2 1420 microplate reader (PerkinElmer, Massachusetts, USA). Relative effects on cell viability were calculated in relation to the vehicle control.

### Statistical analyses

Results from the dual luciferase assay and the viability assay were processed using R and GraphPad Prism 7 (GraphPad Software, La Jolla, USA). Graphs and illustrations were designed using GraphPad Prism 7. For both assays data of three or four experiments (experimental unit n = 3–4), each performed with quadruplicate samples for each concentration, were pooled and log-transformed to achieve homoscedasticity, giving a total population size for every exposure group of 10–16 (observational unit N = 10–16). Data was normalized against internal background within the dual reporter gene assay and against the negative control, giving fold induction as a final output. Significant differences between the control and the exposure groups were analyzed via a mixed-model two way-ANOVA followed by Dunnett’s post-hoc test^28^. Thereby, transformed output data was considered as a fixed factor whereas experiments were considered as a random factorial factor within the model in order to account for inter-experimental variation. A p < 0.05 was considered statistically significant. Normality (quantile-quantile-plot) and homoscedasticity (residuals-vs.-fitted) of data sets were checked via graphical analysis of residuals.

## Results

### Transfection efficiency of various commercial reagents in zebrafish cell lines

Establishment of transgenic cell lines requires knowledge on transfection efficiency of different available transfection reagents for specific cell lines. Most commercially available transfection reagents are developed for transfection of mammalian cells. We have therefore tested the transfection efficiency of twelve commercially available transfection reagents in all three zebrafish cell lines (see supplementary data). The cells were transfected with a Renilla luciferase plasmid in different reagent to DNA mass ratios, according to the manufacturer’s instructions. The total Renilla luminescence was analyzed as a measurement of transfection efficiency. Additionally, increasing cell densities were tested in the best detected DNA to transfection reagent ratios (data not shown). The following transfection parameters were found to result in the highest transfection efficiency for Pac2, ZF4 and ZFL, respectively: Pac2, plating density of 10^4^ cells/well and a 4 µg FHD to 1 µg DNA ratio (Fig. S1). ZF4, plating density of 1.25*10^4^ cells/well and a 2 µg FHD to 1 µg DNA ratio (Fig. S2). ZFL, plating density of 2.5*10^4^ cells/well and a 2 µg XHP to 1 µg DNA ratio or jetPRIME in a 3 to 1 ratio (Fig. S3). Finally, FHD for the Pac2 and ZF4 and XHP for the ZFL cell lines were chosen for further experiments .

### Activation of Nrf2 and cell viability in zebrafish cell lines

All three zebrafish cell lines were co-transfected with an Nrf2-responsive Firefly-luciferase plasmid and a normalizing Renilla-luciferase plasmid. In order to quantify cell line specific responses, cells were exposed to known or postulated Nrf2 inducers tBHQ, SFN, and H_2_O_2_ in concentrations of 0.1 to 100 µM for 24 h. DMSO was used as a cytotoxic control. In parallel to detecting Nrf2-activation via the dual reporter gene assays, cell viability was measured within the same experimental set-up using the MTS-assay.

**Figure 1:**
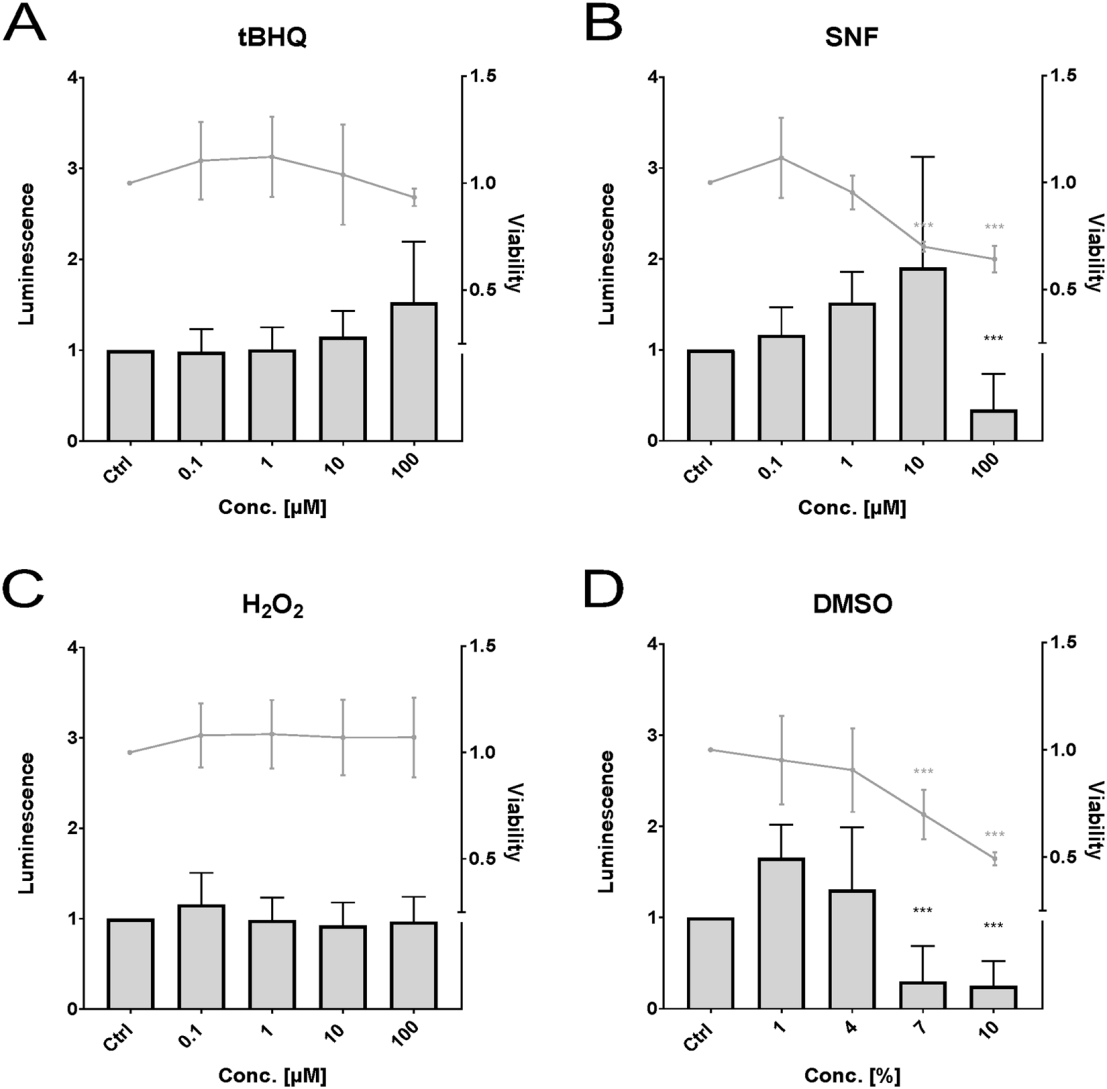
Effects of *tert*butylhydroquinone (**A**, “tBHQ”), sulforaphane (**B**, “SFN”), hydrogen peroxide (**C**), and dimethyl sulfoxide (**D**, “DMSO”) on luminescence (bars) and viability (lines) measured in the zebrafish cell line Pac2. Luminescence corresponds to quantitative Nrf2 activation measured via dual reporter gene assay. Viability corresponds to measured absorbance of formazan product via MTS-assay. Each bar and point respectively represent the mean (experimental units n = 3–4; observational units N = 10–16) including SD. Asterisks indicate significance tested in a two-way ANOVA mixed model with Dunett’s post-hoc test (***p < 0.001; grey = viability; black = luminescence).

In the Pac2 cell line (Fig. 1) there was no statistically significant increase in Nrf2 activity by any of the tested compounds. Statistically significant decrease in activity was observed for SFN (Fig. 1B) at a concentration of 100 µM and for DMSO (Fig. 1D) at 7% and 10%. Statistically significantly reduced cell viability was observed at 10 and 100 µM for SFN and at 7% and 10% for DMSO.

**Figure 2:**
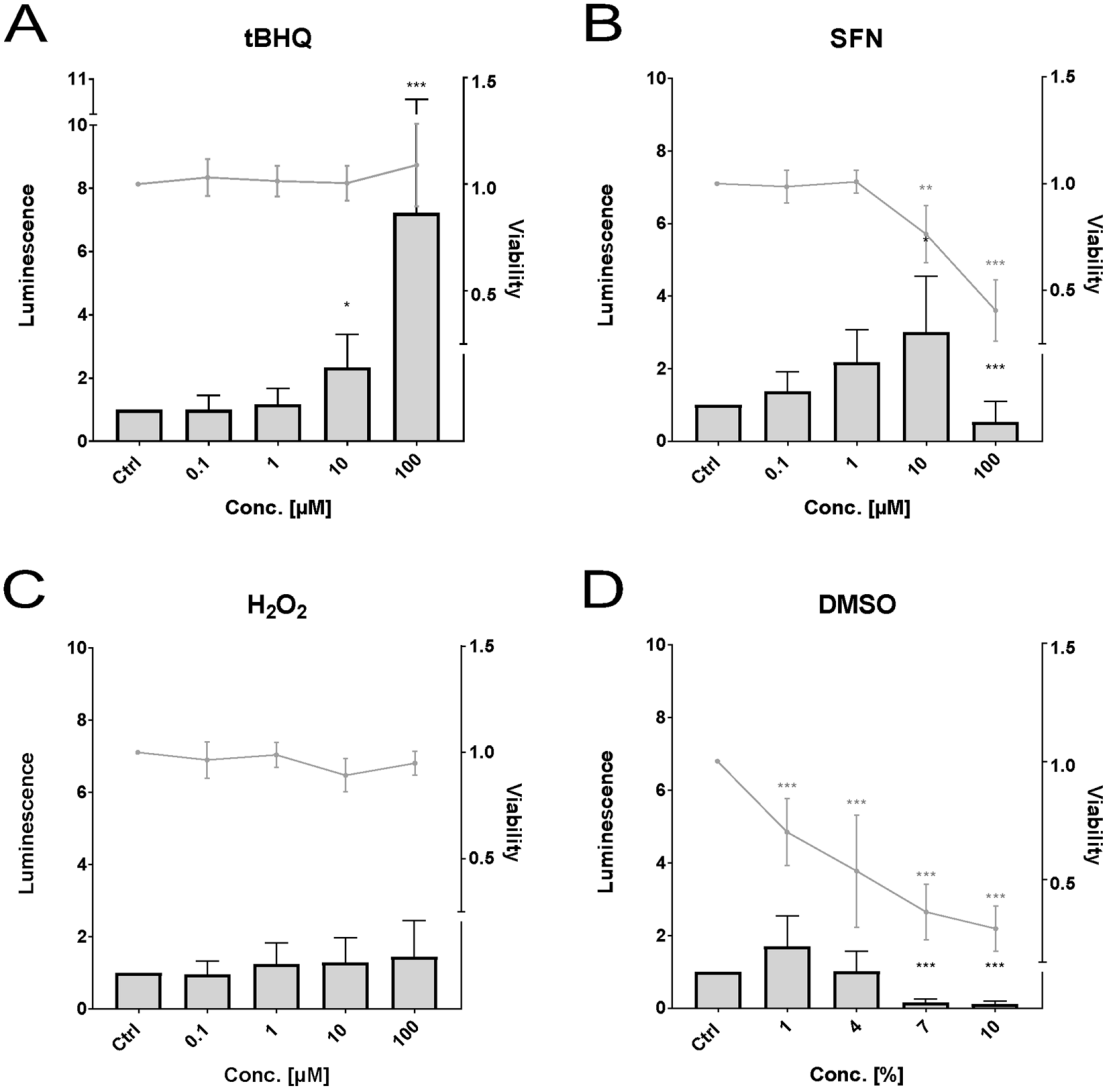
Effects of *tert*butylhydroquinone (**A**, “tBHQ”), sulforaphane (**B**, “SFN”), hydrogen peroxide (**C**), and dimethyl sulfoxide (**D**, “DMSO”) on luminescence (bars) and viability (lines) measured in the zebrafish cell line ZF4. Luminescence corresponds to quantitative Nrf2 activation measured via dual reporter gene assay. Viability corresponds to measured absorbance of formazan product via MTS-assay. Each bar and point respectively represent the mean (experimental units n = 3–4; observational units N = 10–16) including SD. Asterisks indicate significance tested in a two-way ANOVA mixed model with Dunett’s post-hoc test (*p < 0.05, **p < 0.01, ***p < 0.001; grey = viability; black = luminescence).

In the ZF4 cell line (Fig. 2) statistically significant induction of the Nrf2 activity was observed for tBHQ (Fig. 2A) at 10 (2.3-fold) and 100 µM (7.2-fold) and for SFN (Fig. 2B) at 10 µM (3-fold). Statistically significant decrease in activity was observed for SFN (Fig. 2B) at a concentration of 100 µM and for DMSO (Fig. 2D) at 7% and 10%. Significant cytotoxicity was detected for DMSO within all tested concentrations, for SFN at a concentration of 10 µM and 100 µM. H_2_O_2_ (Fig. 2C) did not alter the Nrf2 activity nor the cell viability.

**Figure 3:**
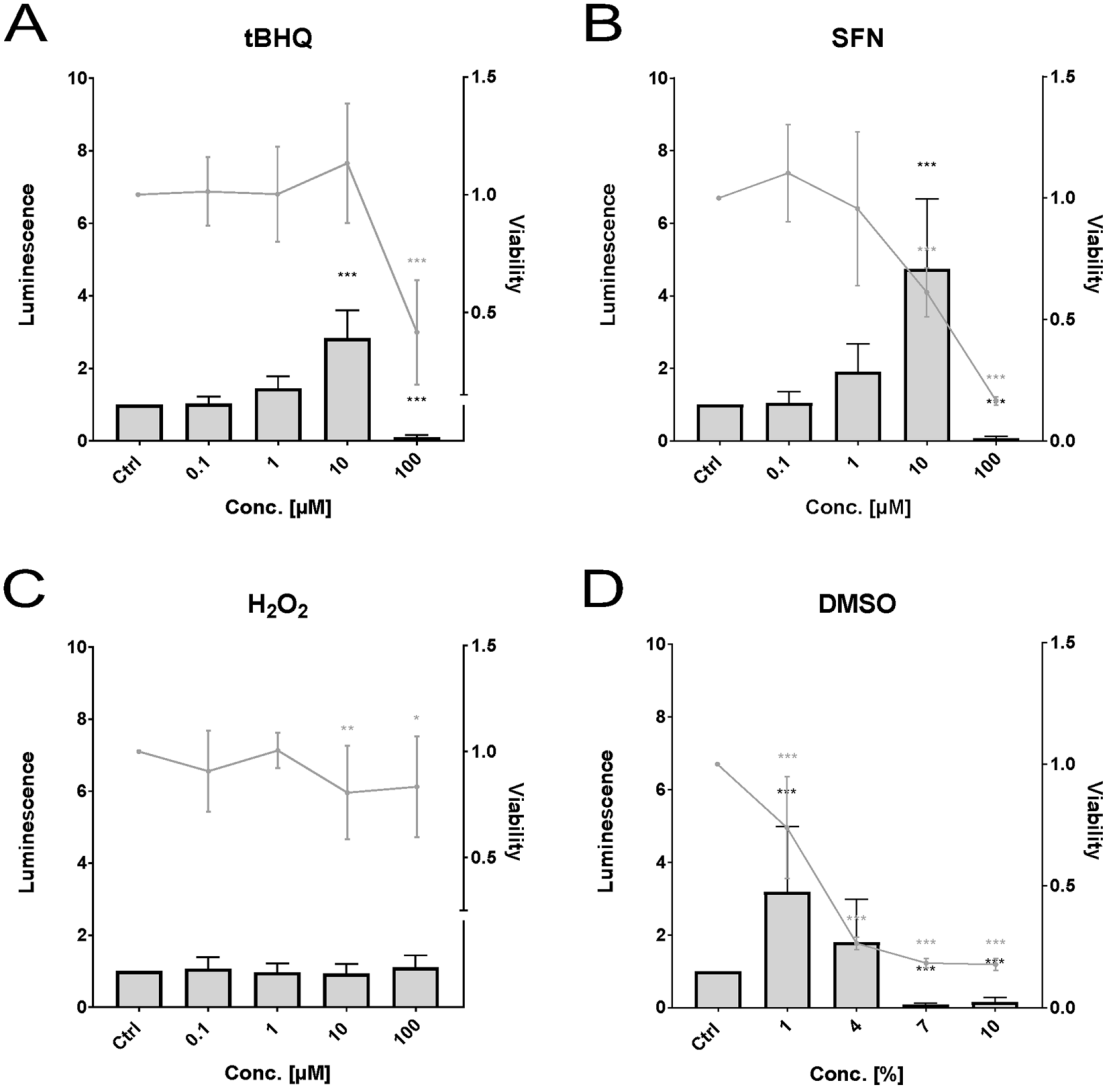
Effects of *tert*butylhydroquinone (**A**, “tBHQ”), sulforaphane (**B**, “SFN”), hydrogen peroxide (**C**), and dimethyl sulfoxide (**D**, “DMSO”) on luminescence (bars) and viability (lines) measured in the zebrafish cell line ZFL. Luminescence corresponds to quantitative Nrf2 activation measured via dual reporter gene assay. Viability corresponds to measured absorbance of formazan product via MTS-assay. Each bar and point respectively represent the mean (experimental units n = 3–4; observational units N = 10–16) including SD. Asterisks indicate significance tested in a two-way ANOVA mixed model with Dunett’s post-hoc test (*p < 0.05, **p < 0.01, ***p < 0.001; grey = viability; black = luminescence).

In the ZFL cell line (Fig. 3) statistically significant activation of Nrf2 was observed for tBHQ (Fig. 3A) at a concentration of 10 µM (2.8-fold), for SFN (Fig. 3B) also at 10 µM (4.7-fold), and for DMSO (Fig. 3D) at 1% (3.2-fold). Statistically significant decrease in activity was observed for tBHQ and SFN at 100 µM and for DMSO at 7% and 10%. SFN showed statistically significant cytotoxicity at 10 and 100 µM, tBHQ at 100 µM, and DMSO at all concentrations. For H_2_O_2_ (Fig. 3C), no statistically significant change in Nrf2 activity was observed, although at 10 µM and at 100 µM a slight cytotoxicity was detected.

### Activation of Nrf2 by pesticides

The cell lines ZF4 and ZFL showed a higher sensitivity to the tested positive controls (section 3.2), as compared to the Pac2 cell line. Therefore, the latter was not further tested since in addition fibroblast-like cells were already covered by ZF4. To test the applicability of the assay, ZF4 and ZFL cells transfected with the Nrf2 sensitive luciferase plasmid were used to investigate the potential oxidative stress potency of six pesticides.

**Figure 4:**
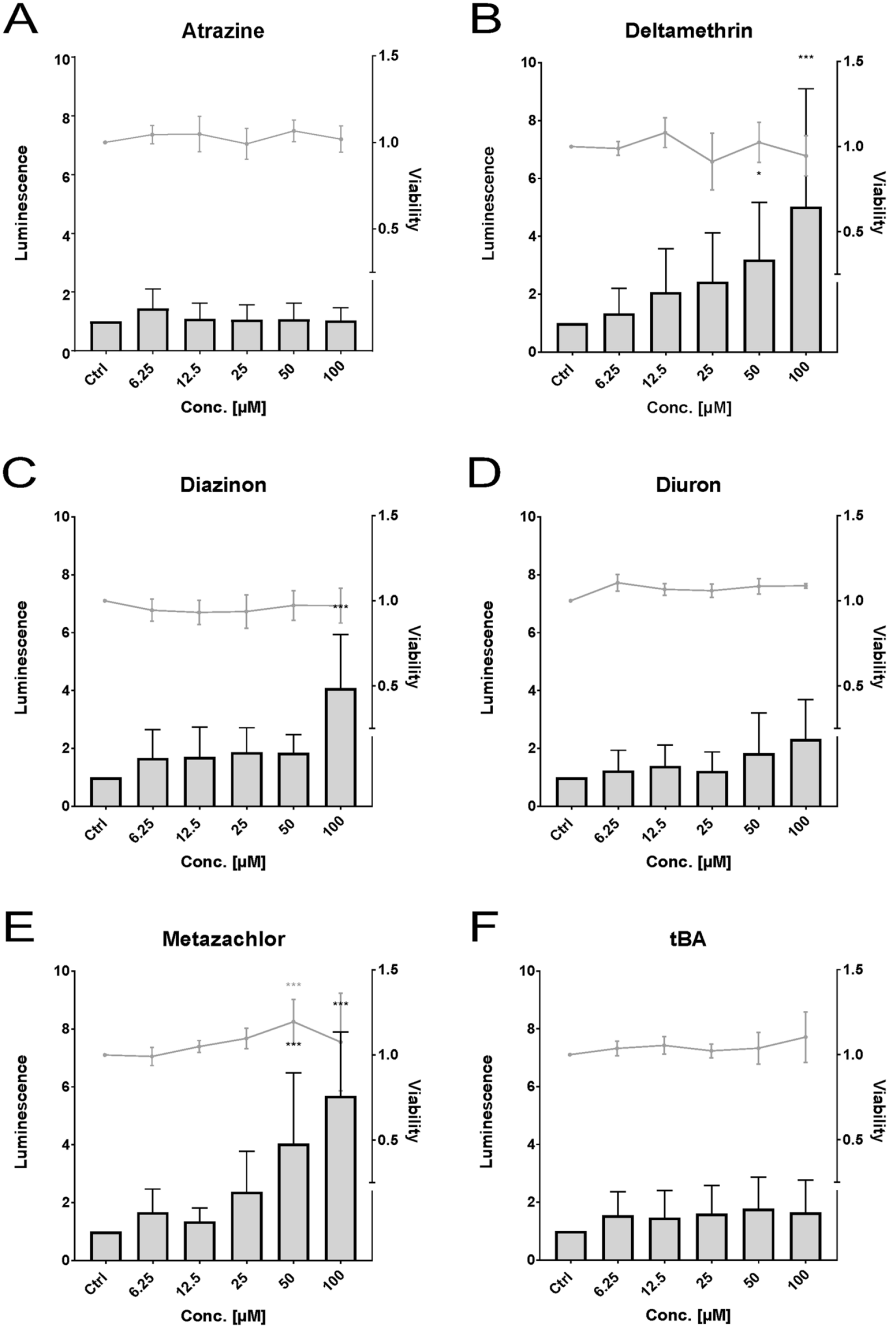
Effects of the pesticides atrazine (**A**), deltamethrin (**B**), diazinon (**C**), diuron (**D**), metazachlor (**E**), and terbutylazine (**F**, “tBA”) on luminescence (bars) and viability (lines) measured in the zebrafish cell line ZF4. Luminescence corresponds to quantitative Nrf2 activation measured via dual reporter gene assay. Viability corresponds to measured absorbance of formazan product via MTS-assay. Each bar and point respectively represent the mean (experimental units n = 3–4; observational units N = 10–16) including SD. Asterisks indicate significance tested in a two-way ANOVA mixed model with Dunett’s post-hoc test (*p < 0.05, ***p < 0.001; grey = viability; black = luminescence).

In the ZF4 cell line, we found that the Nrf2 activity was significantly increased by the pesticides deltamethrin (Fig. 4B) at 50 µM (3.2-fold) and 100 µM (5-fold), diazinon (Fig. 4C) at 100 µM (4.1-fold), and metazachlor (Fig. 4E) at 50 µM (4-fold) and 100 µM (5.7-fold). No effects on the Nrf2 activity were observed after atrazine, diuron and terbutylazine exposure (Fig. 4A). No cytotoxicity was observed for any of the pesticides.

**Figure 5:**
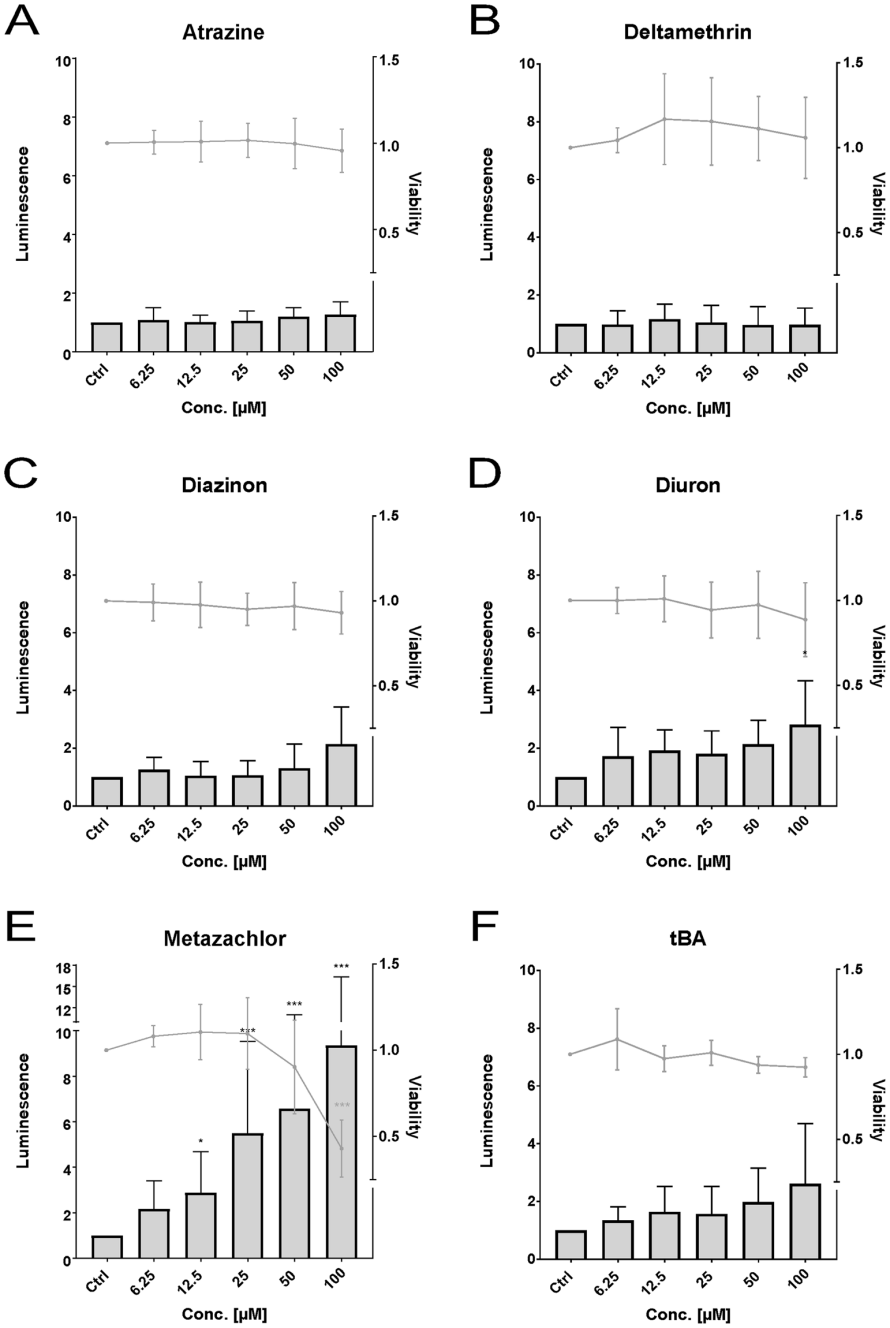
Effects of the pesticides atrazine (**A**), deltamethrin (**B**), diazinon (**C**), diuron (**D**), metazachlor (**E**), and terbutylazine ((**F**, “tBA”) on luminescence (bars) and viability (lines) measured in the zebrafish cell line ZFL. Luminescence corresponds to quantitative Nrf2 activation measured via dual reporter gene assay. Viability corresponds to measured absorbance of formazan product via MTS-assay. Each bar and point respectively represent the mean (experimental units n = 3–4; observational units N = 10–16) including SD. Asterisks indicate significance tested in a two-way ANOVA mixed model with Dunett’s post-hoc test (*p < 0.05, ***p < 0.001; grey = viability; black = luminescence).

In the ZFL cell line, statistically significant Nrf2 activation was observed for diuron (Fig. 5D) (2.8- fold) at 100 µM and for metazachlor (Fig. 5E) at 12.5 µM (2.9-fold), 25 µM (5.5-fold), 50 µM (6.6-fold), and 100 µM (9.3-fold). Atrazine (Fig. 5A), deltamethrin (Fig. 5B), diazinon, and terbutylazine did not induce any effects, neither on the Nrf2 activity nor the cell viability. A statistically significant decrease in viability was observed for metazachlor at 100 µM, while all other groups showed no effects on cell viability.

## Discussion

The Keap1-Nrf2-ARE signaling pathway is a major balancer of oxidative homeostasis, given its role in oxidative stress response but also physiological regulation^29^. Oxidative stress may lead to toxicity *e.g*. via enzyme deactivation, lipid peroxidation, and DNA damage. These effects may lead to tissue damage, apoptosis or cell necrosis^1,30^ and further to developmental toxicity^31,32^ and cancer^33^. So far, *in vivo* methods for determination of Nrf2 activity have been established in zebrafish strains^34–36^, *in vitro* methods using mammalian cells are described^37–40^, and standardized high-throughput assays in human cell lines^41^ have been developed. Here, we report the development of a reporter gene assay for oxidative stress response in zebrafish cell lines.

### Transfection efficiency

In this study, we have tested the transfection efficiency of twelve commercially available transfection reagents in three different cell lines and found a high variability in efficiency between the transfection reagents. We found that FuGENE HD (Promega) showed the highest transfection efficiency in both Pac2 and ZF4, while X-tremeGENE HP (Roche) and jetPRIME (Polyplus) showed the highest transfection efficiency in ZFL. These are important findings for future efforts to establish *in vitro* assays using transfected zebrafish cell lines.

### The oxidative stress response varies within different zebrafish cell lines

We have analyzed the Nrf2 activity in three zebrafish cell lines; Pac2, ZF4 and ZFL. The Pac2 and ZF4 cell lines are pooled embryonic fibroblasts, whereas the ZFL cell line was established from hepatocytes derived from a single adult individual. In this way, different developmental stages and tissue types are represented in the study. Hepatocytes were chosen since the liver is the primary location of detoxification and show highest abundancy in critical phase II enzymes, such as GSTs^42^. In order to test single cell line feasibility, each transfected cell line was first exposed to postulated Nrf2 inducers. The results showed that the Nrf2 activity following exposure varied between cell lines. The Keap1-Nrf2-ARE signaling pathway is not functional immediately after fertilization and the responsiveness to oxidative stress seems to vary during embryogenesis and larval development^32,43,44^. Within 24 hpf, the response to oxidative stress or Nrf2 inducers is quite low, increases up to 48 hpf, exhibits high variability during hatching and post-hatching, and finally stabilizes at around 96 hpf. This could be an explanation for the observed differences in sensitivity between the three investigated cell lines, as they are derived from zebrafish in different developmental stages. In addition, an impact of total serum concentration in the culture medium and therefore less available, non-protein bound compound concentration could be an alternative explanation for the differences in response between these zebrafish cell lines. Pac2 cells are cultured at a higher serum concentration (15%) than ZF4 and ZFL cells (10 and 5% respectively), which might explain the observed differences in sensitivity. Since Nrf2 is ubiquitously expressed in most cell types and acts via a common mechanism, its activity is generally only assayed in a single cell type when using bioassays based on mammalian cells^45–47^. However, our result show that cell line selection is crucial for this assay and further research is needed to fully explain the observed differences in oxidative stress sensitivity in zebrafish cell lines.

### H_2_O_2_ does not activate Nrf2 in zebrafish cell lines

Surprisingly, we were unable to show an Nrf2 induction by H_2_O_2_ in any of the cell lines studied, although H_2_O_2_ is normally metabolized by GSTs which are Nrf2 regulated. However, Nrf2-recruitment failure by H_2_O_2_ has previously been described during development in embryos^35,48,49^ and in larvae^36^. The phenomenon is either interpreted as a systemic breakdown of the Nrf2-Keap1 axis^48^ or absence of an intermediate “posthatch factor” which is induced by H_2_O_2_ and then modulates the Nrf2 response^35^. On the contrary, Nrf2 induction by H_2_O_2_ was reported in mammalian cell lines^50^. Species differences, methodological aspects, since H_2_O_2_ is a very reactive compound with short half-life, and interactions with media components may partly explain why we did not see any Nrf2 induction by H_2_O_2._

### Application of an Nrf2 responsive reporter gene assay

To test the potential application of the established assay, six pesticides suspected to cause oxidative stress^22^ were analyzed. We found that four out of the six analyzed compounds significantly increased the Nrf2 activity in at least one cell line. The dose-effect relationship for the compounds differed, where some compounds showed a clear dose-dependent increase in the Nrf2 activity, while other compounds showed a threshold-like effect with an increased Nrf2 activity only observed within the highest exposure concentration. However, those compounds would need to be tested at higher, non-toxic concentrations in order to exclude dose-dependency. These different patterns may be explained by differing mechanisms by which the compounds are inducing the Nrf2 release from Keap1 and thereby trigger the oxidant stress response. Such a mechanism-related model has been proposed by Kobayashi, Yamamoto, and co-workers^9,35,51,52^.

It has been reported that deltamethrin is causing alterations in antioxidant enzymes (glutathione S-transferase (GST), superoxide dismutase (SOD), catalase (CAT), glutathione peroxidase (GPx), and glutathione reductase (GR)), reduced glutathione concentration levels (GSH), and lipid peroxidation levels (LPO) in Prussian carp (*Carassius gibelio*)^53,54^, spotted snakehead (*Channa punctata*)^55,56^, carp (*Cyprinus carpio)*^57^, and bushynose (*Ancistrus multispinis*)^58^, whereas other species did not show a similar response to deltamethrin^59^. We report an effect in ZF4, but no effects were observed in ZFL. Deltamethrin is primarily metabolized by phase I enzymes CYP1A1, CYP1A2, and carboxylesterases^60,61^. The lack of effect in the ZFL cell line may therefore be due to a faster metabolism of the compound within hepatocytes, since functional phase I activity has been reported *in vitro*^27,62,63^ on transcript and protein levels, although also other mechanisms might explain the observed difference in response in ZFL and ZF4. Alterations in antioxidant enzymes, GSH, and LPO levels have also been reported in carp (*Cyprinus carpio)* after exposure to diazinon^64,65^. Beyond that, histopathological endpoints had been identified in bluegill sunfish *(Lepomis macrochirus*)^66^. Diuron has been reported to induce oxidative stress after chronic exposure to goldfish (*Carassius auratus*)^67^, alternating GST and CAT levels in tropical fish (*Astyanax* sp.)^68^, and genotoxicity in rainbow trout liver and gill cell lines (RTL-W1, RTG-W1)^69^. An induced Nrf2 activity by diuron and diazinon has also been reported in human liver hepatoma cell line (HepG2) at comparable concentrations^11^. Metazachlor was the most potent inducer of Nrf2 activity, showing a higher potency than the known inducers used as positive controls. No studies on oxidative stress response by metazachlor were found in the literature. However, regulatory toxicity testing has demonstrated low acute toxicity in rainbow trout (Oncorhynchus mykiss), carp, and bluegill sunfish^70^. In mammals limited evidence of carcinogenic effects has been reported besides acute toxicity^71^. To our knowledge, this is a novel finding on the toxicity of metazachlor in vertebrates and prompts further investigation regarding the toxicity of metazachlor.

## Conclusion

We describe the development and application of a novel Nrf2 responsive reporter gene assay to monitor oxidative stress response in various zebrafish cell lines. Further, we have shown that the developed assay is useful for chemical testing by analyzing the effect of postulated Nrf2 inducers and compounds which were suggested to induce oxidative stress in fish. We suggest that this model could be a valuable tool for future research in aquatic toxicology to study the toxicity of both pure compounds and environmental samples.

## Electronic supplementary material

Supplementary Information

## Data Availability

The datasets generated during the current study are available from the corresponding author upon request.
