# Supplementary material for: Development of an oxidative stress in vitro assay in zebrafish (Danio rerio) cell lines
Source: Sci Rep. 2018 Aug 17;8:12380. doi: 10.1038/s41598-018-30880-1 (PMC6098050; doi:10.1038/s41598-018-30880-1)
Supplement: Supplementary file 1 — Supplementary Information [file 41598_2018_30880_MOESM1_ESM.pdf]

Development of an oxidative stress *in vitro* assay in zebrafish (*Danio rerio*) cell lines

SUPPLEMENTARY INFORMATION

Sebastian Lungu-Mitea*, Agneta Oskarsson, and Johan Lundqvist

Department of Biomedicine and Veterinary Public Health, Swedish University of Agricultural Sciences, Box 7028, SE-750 07 Uppsala, Sweden

* Corresponding author:
Sebastian Lungu-Mitea
E-mail: sebastian.lungu@slu.se

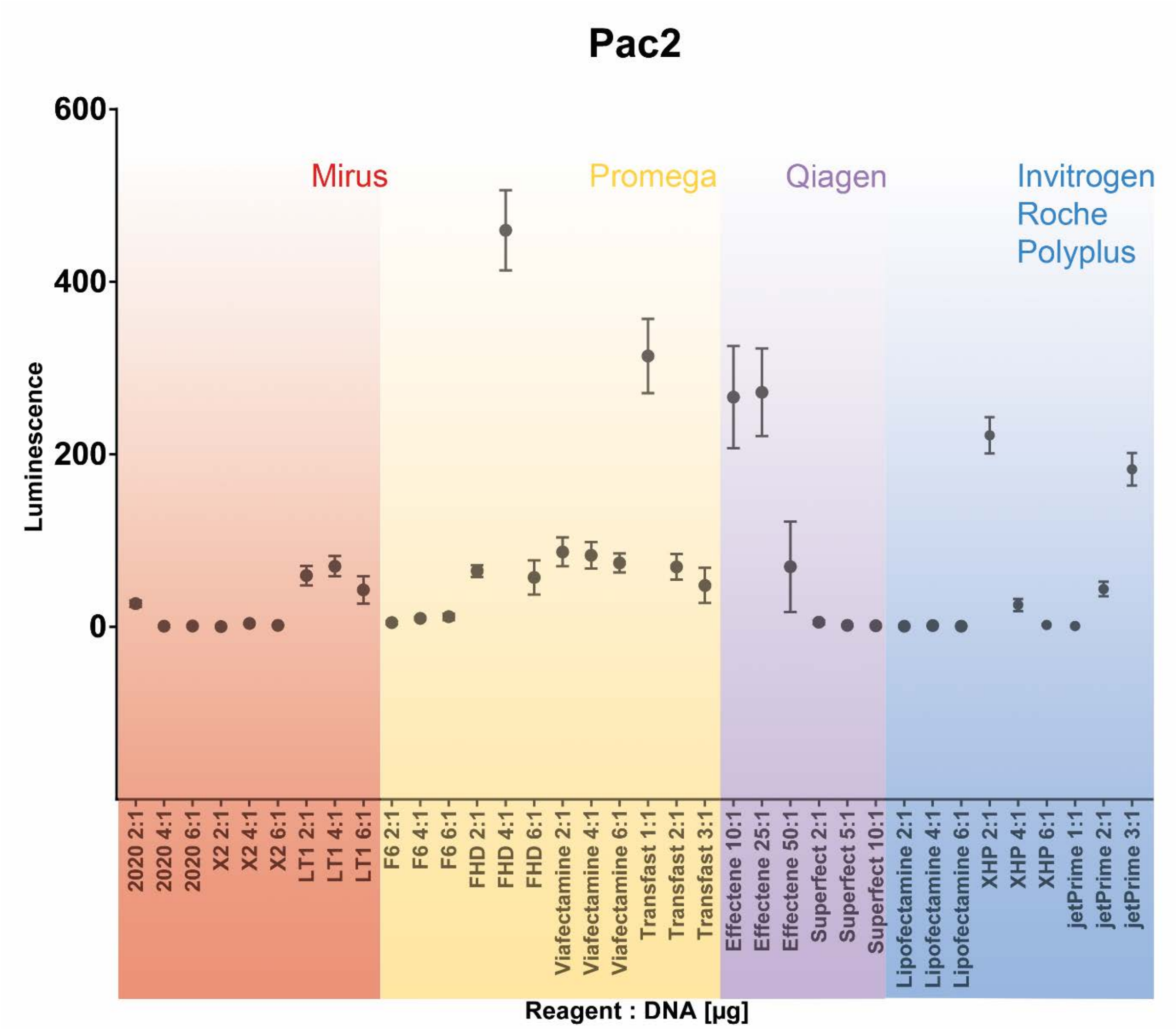

Fig. S1: Transfection efficiency in the zebrafish cell line Pac2. Cells were transfected in quadruplicates in different reagent to DNA mass ratios using 12 transfection reagents (see color-code for specific producer). Efficiency corresponds to measured luminescence in a reporter gene assay using transient Renilla luciferase background induction only, without normalization. Points and whiskers represent mean + SEM (n = 4).

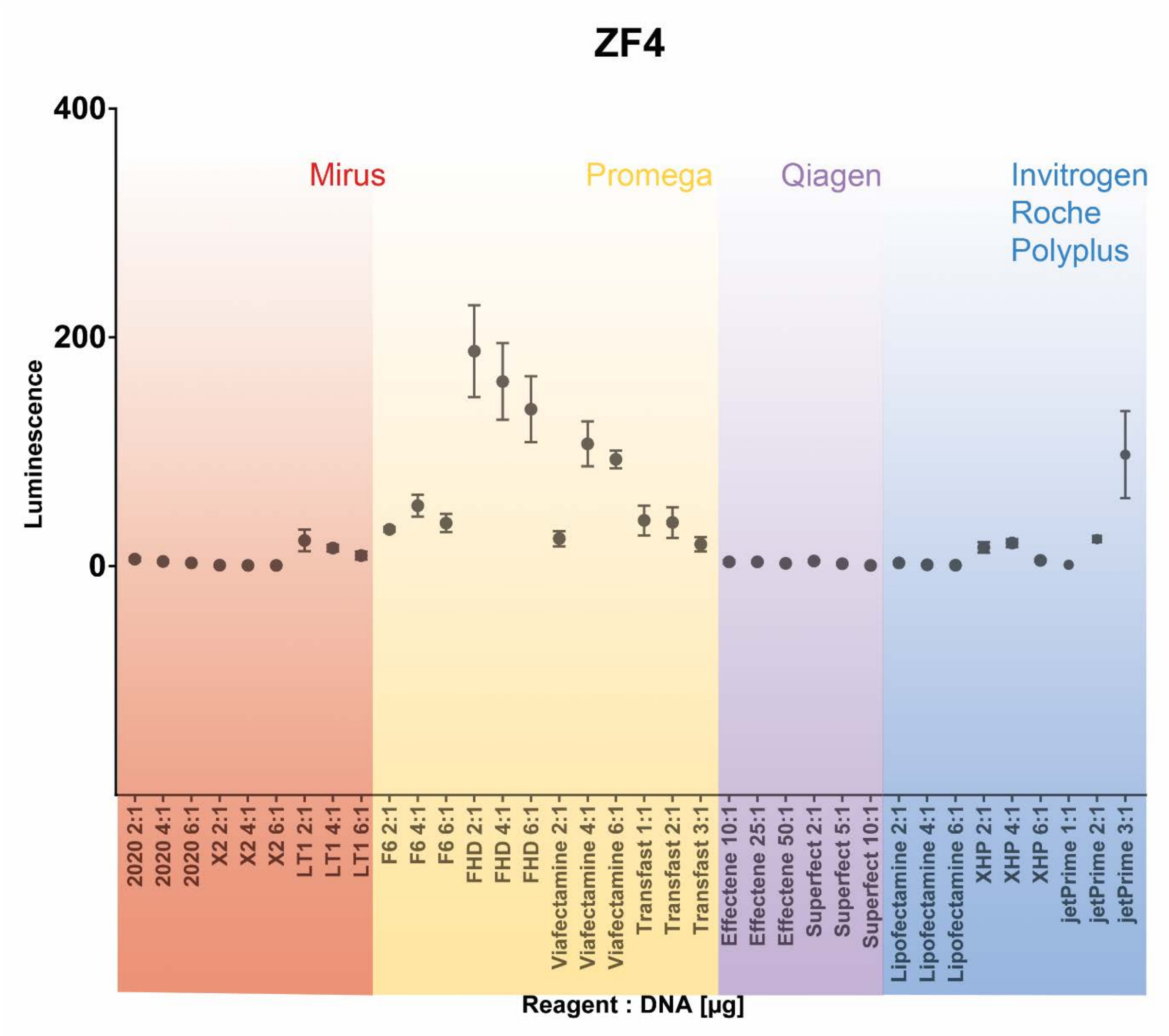

Fig. S2: Transfection efficiency in the zebrafish cell line ZF4. Cells were transfected in quadruplicates in different reagent to DNA mass ratios using 12 transfection reagents (see color-code for specific producer). Efficiency corresponds to measured luminescence in a reporter gene assay using transient Renilla luciferase background induction only, without normalization. Points and whiskers represent mean + SEM (n = 4).

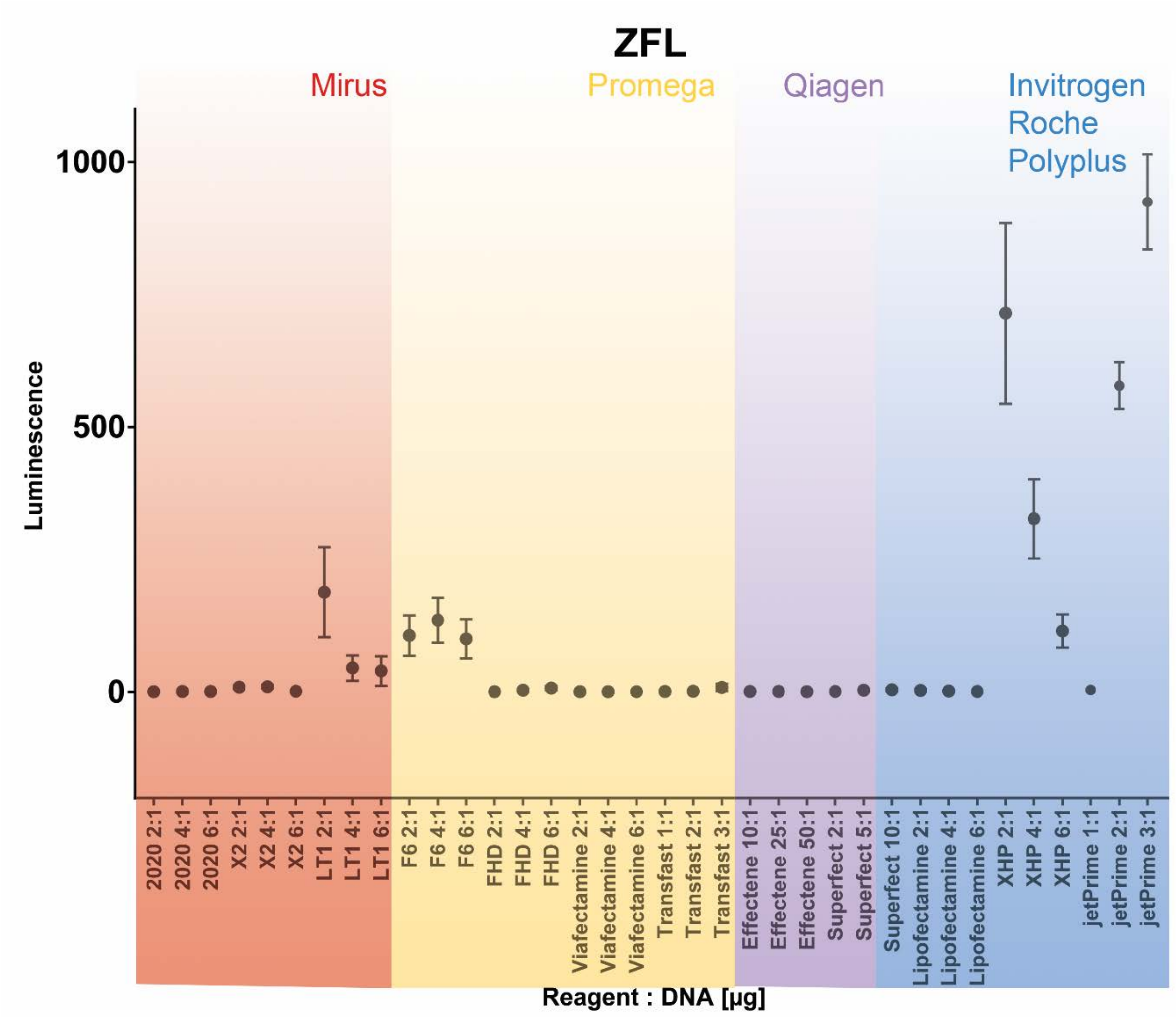

Fig. S3: Transfection efficiency in the zebrafish cell line ZFL. Cells were transfected in quadruplicates in different reagent to DNA mass ratios using 12 transfection reagents (see color-code for specific producer). Efficiency corresponds to measured luminescence in a reporter gene assay using transient Renilla luciferase background induction only, without normalization. Points and whiskers represent mean ± SEM (n = 4).
